# Supplementary material for: Multiplicative joint coding in preparatory activity for reaching sequence in macaque motor cortex
Source: Nat Commun. 2024 Apr 11;15:3153. doi: 10.1038/s41467-024-47511-1 (PMC11009282; doi:10.1038/s41467-024-47511-1)
Supplement: Supplementary file 1 — Supplementary Information [file 41467_2024_47511_MOESM1_ESM.pdf]

Supplementary Figures

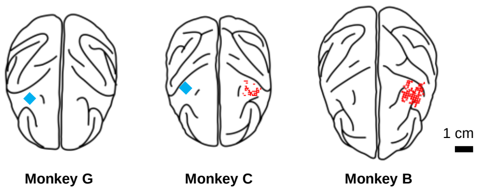

Supplementary Figure 1 Recording locations.

For monkey G, a 96-channel Utah array was implanted in M1 in the right hemisphere. For monkey C, single-electrode recordings were made from his left hemisphere first. Then a 128-channel Utah array was implanted in M1 of its right hemisphere, along with a switch of hands. For monkey B, recording sites for single electrodes were located in the left hemisphere. The recorded hemisphere in all monkeys was contralateral to the hand used during the task.

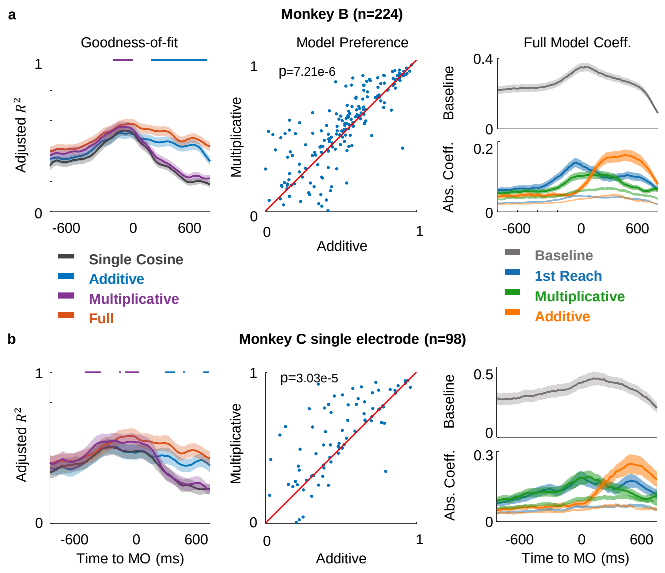

Supplementary Figure 2 Regression performance and coefficient dynamics of single recording.

The same convention as Fig. 5, **a.** Single-electrode recorded data from monkey B. Left: Averaged adjusted R^2 for all fitting models in sliding windows. The upper line shows the significance (Wilcoxon rank sum test, p<0.0005) of comparison between performance of multiplicative (purple line) and additive (blue line) model. Middle: Scatters plot of adjusted R^2 shows that the multiplicative model performed better than the additive model at MO (-100~100ms to MO). Right: Absolute value of each coefficient is averaged across neurons (with twice standard error shaded), the temporal dynamics of which shows the contribution of terms. The coefficient weight of permutation test was plotted in light shade as the chance level. **b.** The results of single electrode recording from monkey C. The time-evolving patterns of model performance are similar among monkeys.

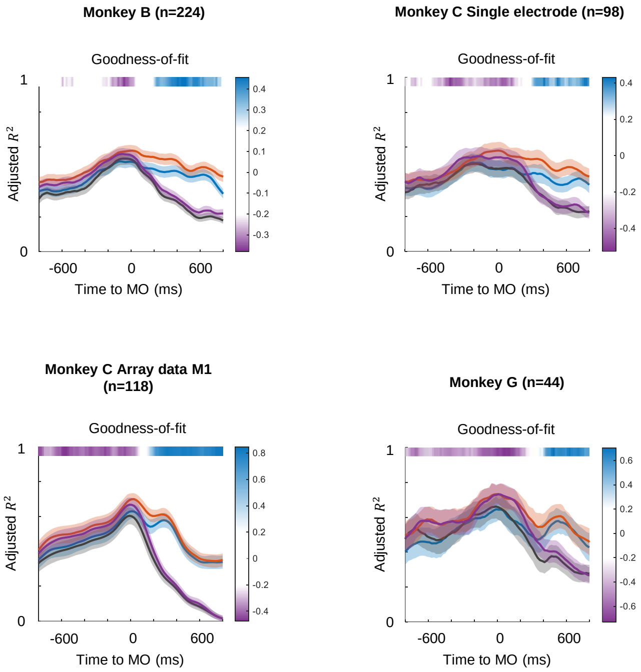

Supplementary Figure 3 Dynamics of goodness-of-fit and effect size

Results of regression are illustrated at the population level for each dataset. Goodness-of-fit was evaluated with averaged adjusted R^2 for all fitting models in a sliding window (with twice the standard error in shade). The upper line shows the effect size r (see Methods) of comparison between performance of the multiplicative and additive models. When r<0, the line is purple, and the multiplicative is superior to additive model; otherwise, blue indicates that the additive model is better.

Supplementary Figure 4 Scatter of goodness-of-fit comparison between multiplicative and additive models of all datasets

Scatters compared the adjusted R^2 between the multiplicative and additive models in 200ms time windows, with p-values indicating the significant difference level of each subplot (Wilcoxon rank sum test). Each ach dot represents the result of a neuron (some neurons may not show in some periods because of no response).

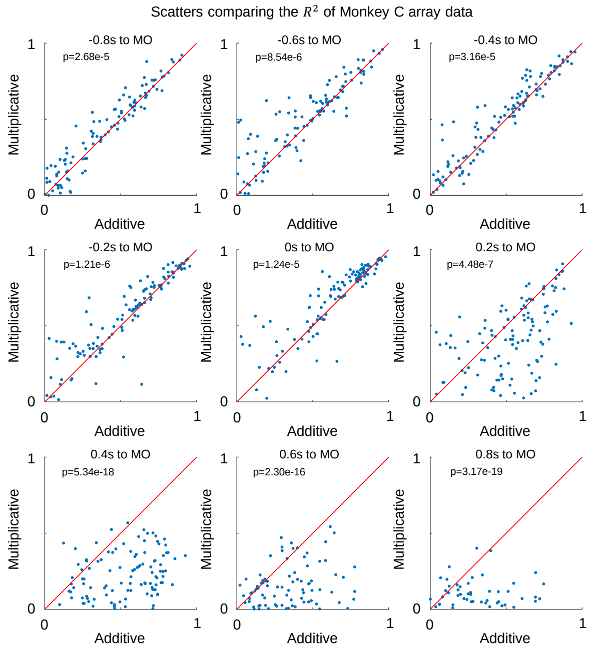

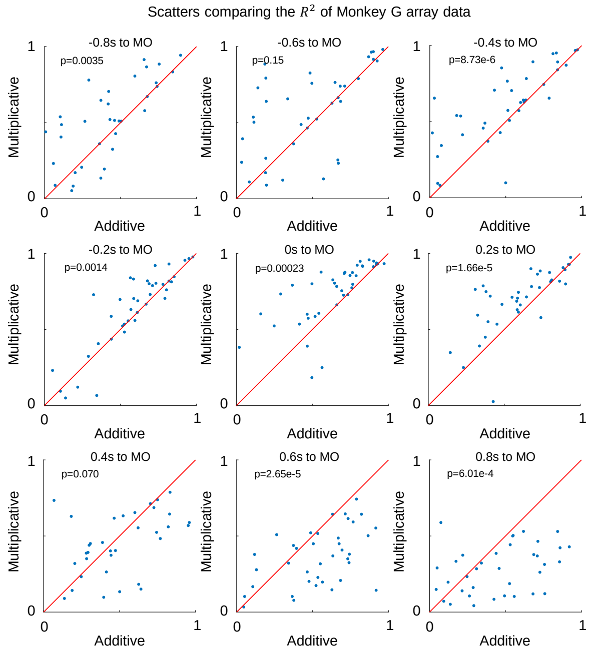

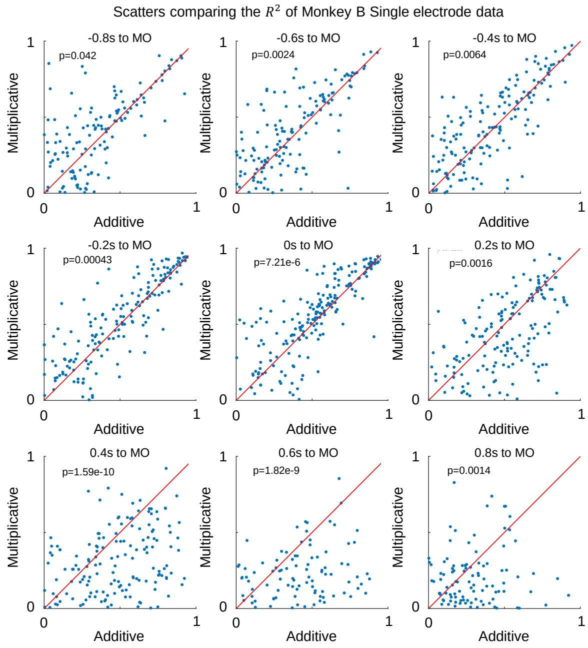
Scatters comparing the R^2 of Monkey B Single electrode data
-0.8s to MO
p=0.042
-0.6s to MO
p=0.0024
-0.4s to MO
p=0.0064
-0.2s to MO
p=0.00043
0s to MO
p=7.21e-6
0.2s to MO
p=0.0016
0.4s to MO
p=1.59e-10
0.6s to MO
p=1.82e-9
0.8s to MO
p=0.0014
Multiplicative
Additive

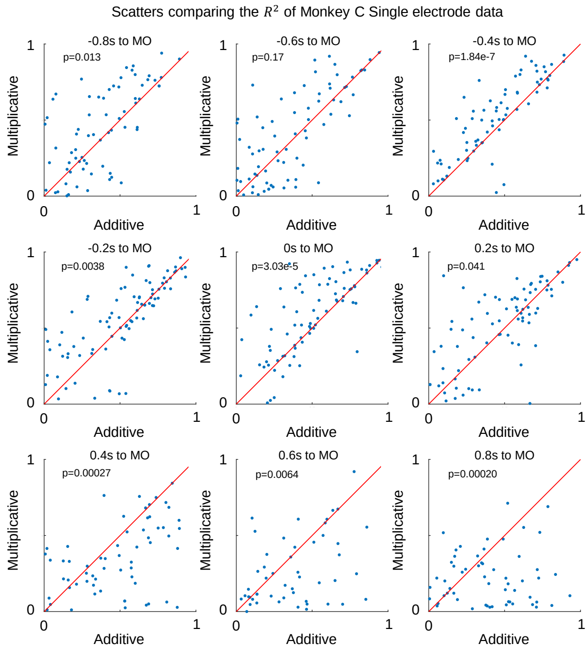
Scatters comparing the R^2 of Monkey C Single electrode data
-0.8s to MO
p=0.013
-0.6s to MO
p=0.17
-0.4s to MO
p=1.84e-7
-0.2s to MO
p=0.0038
0s to MO
p=3.03e-5
0.2s to MO
p=0.041
0.4s to MO
p=0.00027
0.6s to MO
p=0.0064
0.8s to MO
p=0.00020
Multiplicative
Additive

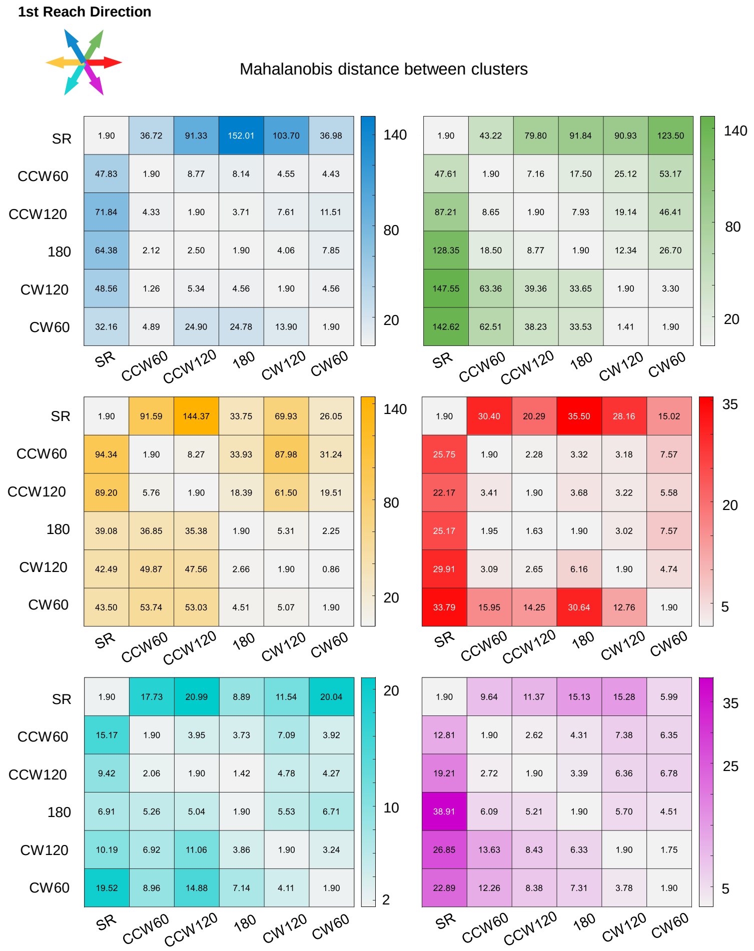

Supplementary Figure 5 Mahalanobis distance of initial state space for each reach direction.

Mahalanobis distance of the initial state projection in Fig.6c. Rows are the observation clusters and columns are the reference clusters. Color represents the 1st reach direction, as in Fig.6.

Supplementary Figure 6 Projection of preparatory activity onto PCA-LDA based initial state space and the mahalanobis distance of all datasets

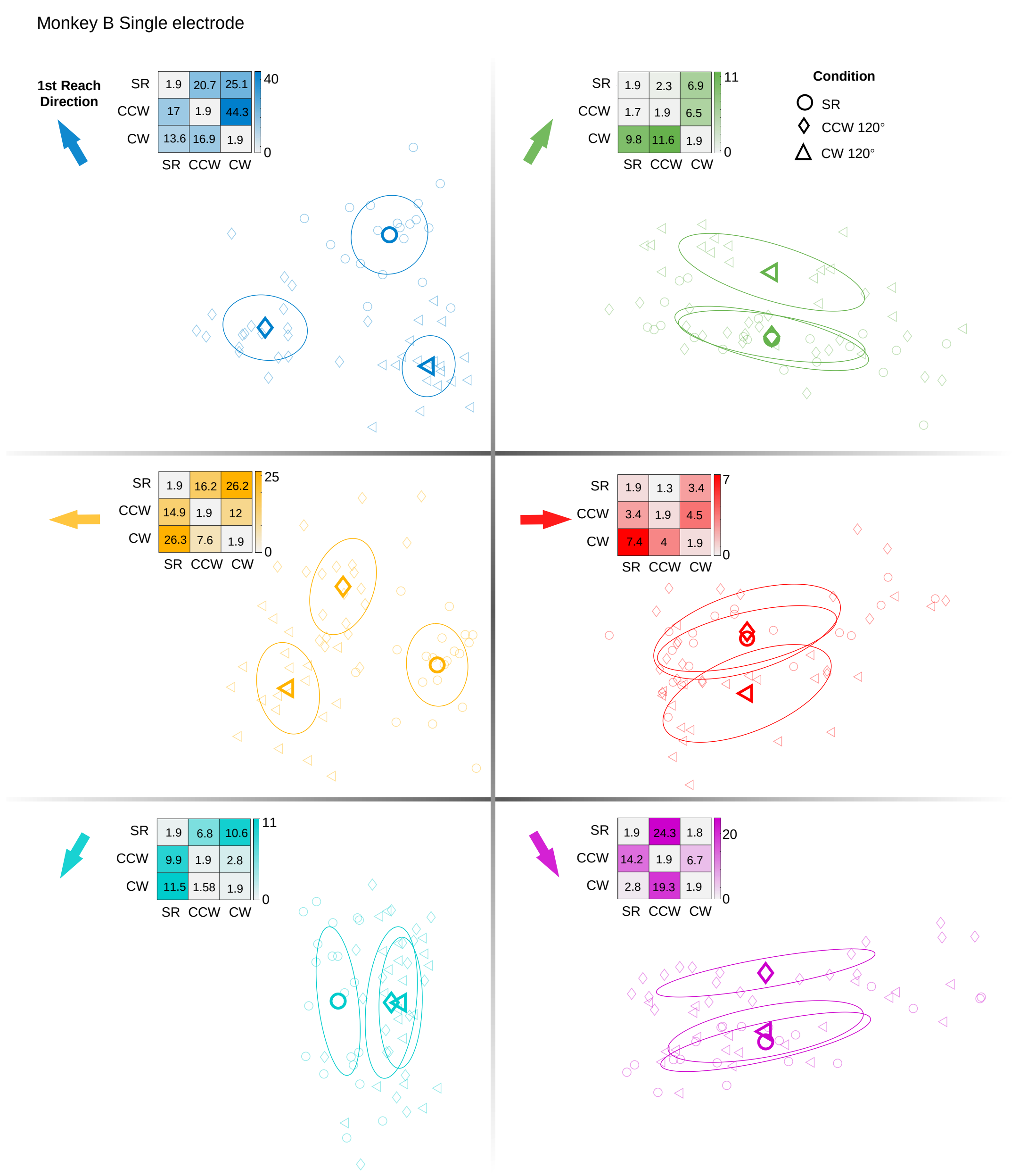

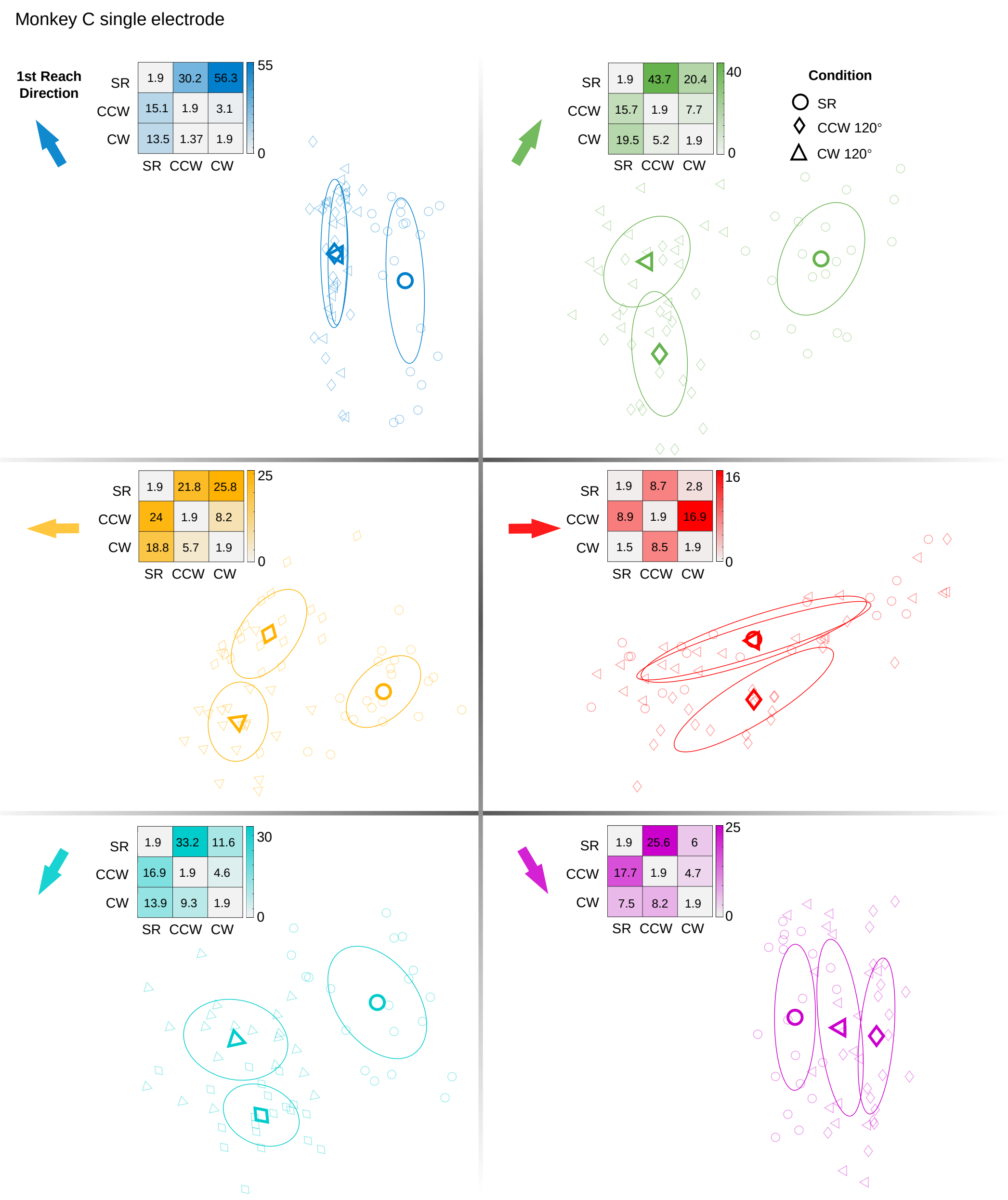
Monkey C single electrode
1st Reach Direction
Condition
SR
CCW 120°
CW 120°
SR CCW CW
1.9 30.2 56.3
15.1 1.9 3.1
13.5 1.37 1.9
55
0
1.9 43.7 20.4
15.7 1.9 7.7
19.5 5.2 1.9
40
1.9 21.8 25.8
24 1.9 8.2
18.8 5.7 1.9
25
1.9 8.7 2.8
8.9 1.9 16.9
1.5 8.5 1.9
16
1.9 33.2 11.6
16.9 1.9 4.6
13.9 9.3 1.9
30
1.9 25.6 6
17.7 1.9 4.7
7.5 8.2 1.9
25

Monkey G Array

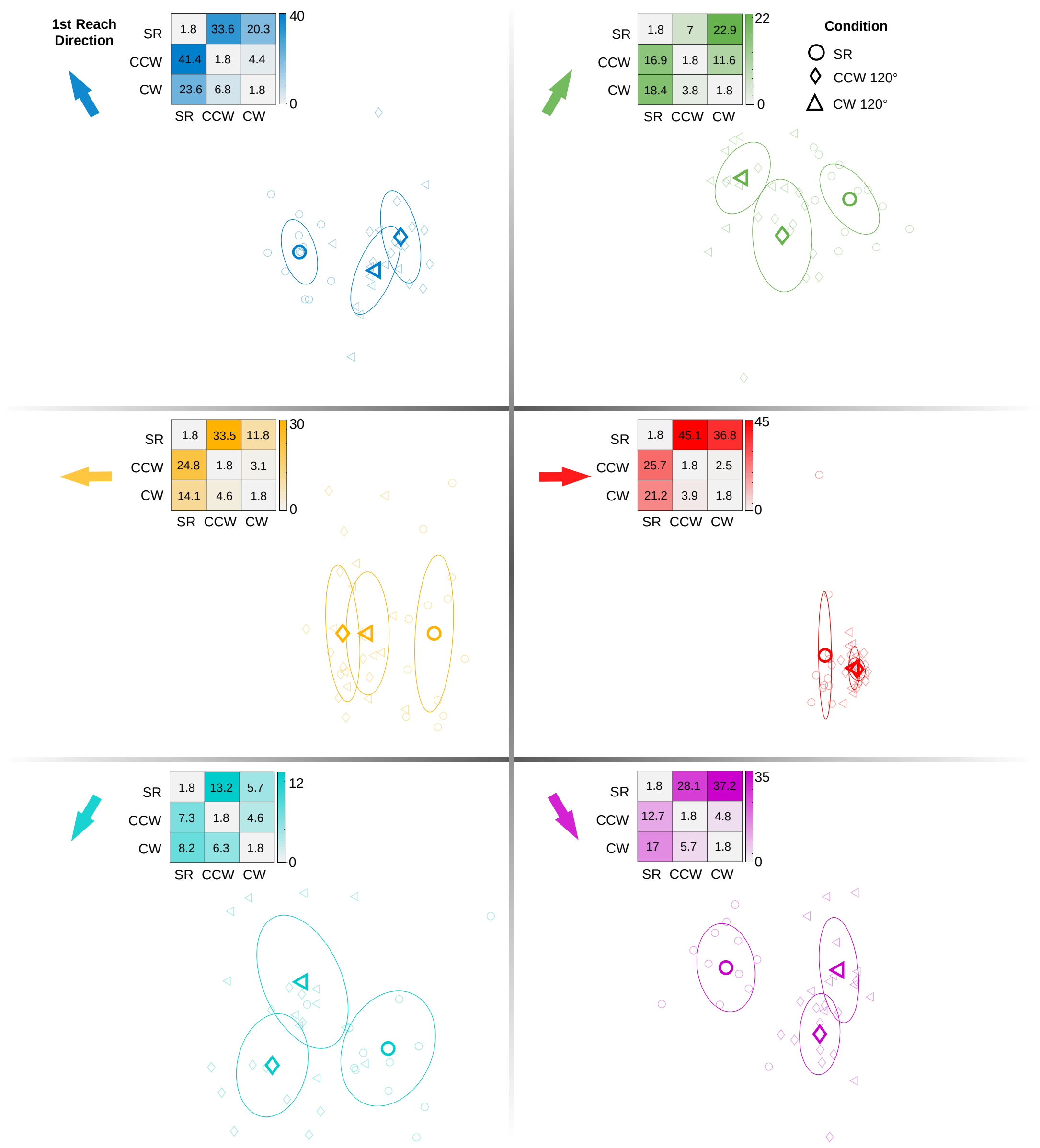

We performed the same subspace analyses as Zimnik & Churchland. The process to identify the preparatory and execution dimensions is as following: First, soft-normalized firing rate of each neuron; Second, mean-centered the firing rate of each neuron within each set of conditions of the same duration (for example, single reaches, compound reaches). They included a ‘delay-period’ epoch 700–150 ms before the onset of single reaches, and the delay-period epoch before compound reaches and delayed double reaches, and also an additional ‘second-reach preparatory’ epoch: 300–150 ms before the onset of the second reach for a preparation-only activity. Execution-only activity was obtained from a peri-reach epoch, −50 to 500 ms relative to reach onset included single reaches, both reaches for delayed double reaches and second reach of compound reaches. The neural dimension was reduced to 20 components, and visualization based on a rotation matrix results from the PCA of 200-ms window of activity before reach onsets. Since there wasn’t a delayed double reach design in our task, we only use the single reach activity to build the preparation and execution subspace, and then project the double-reach activity into those spaces. This change in methodology should make the analysis more rigorous by avoiding building the second reach information into the subspace. This change is better for answering the question of whether both targets can be decoded from the preparatory subspace.

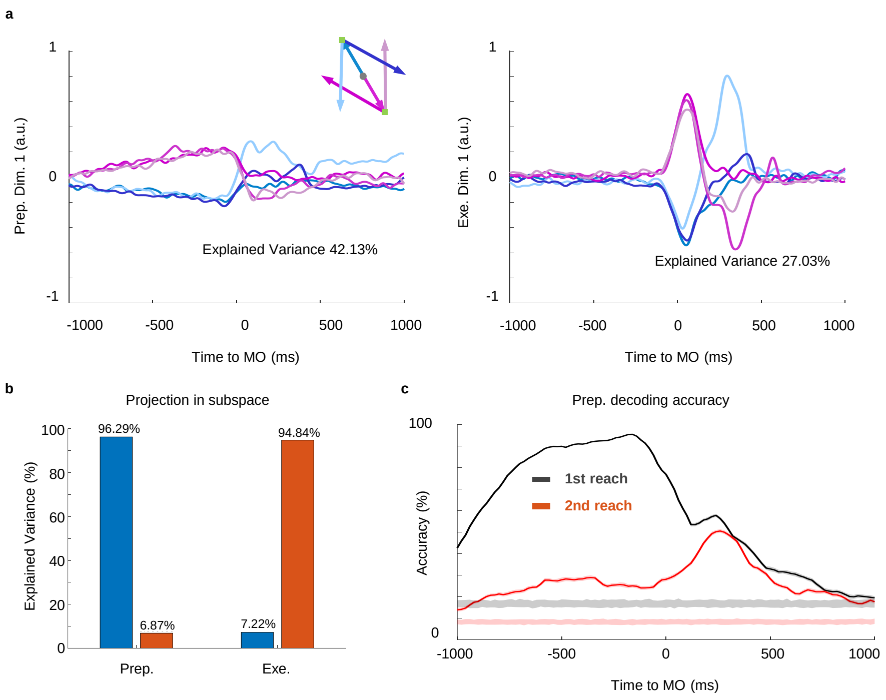

Supplementary Figure 7 Preparatory and execution subspace analysis

a. Projection of single and double reaches onto the first preparatory and execution dimension. Data from monkey C array.

b. Explained variance of projections in preparatory and execution subspaces and vice versa (Elsayed et al., 2016).
c. Decoding accuracy for both reach directions in double reach trials using Prep. Subspace projected data. The decoding accuracy is plotted as a solid line with shade (mean±std). Trial conditions shuffled 100 times to calculate permutation levels and plotted in light shade curves.

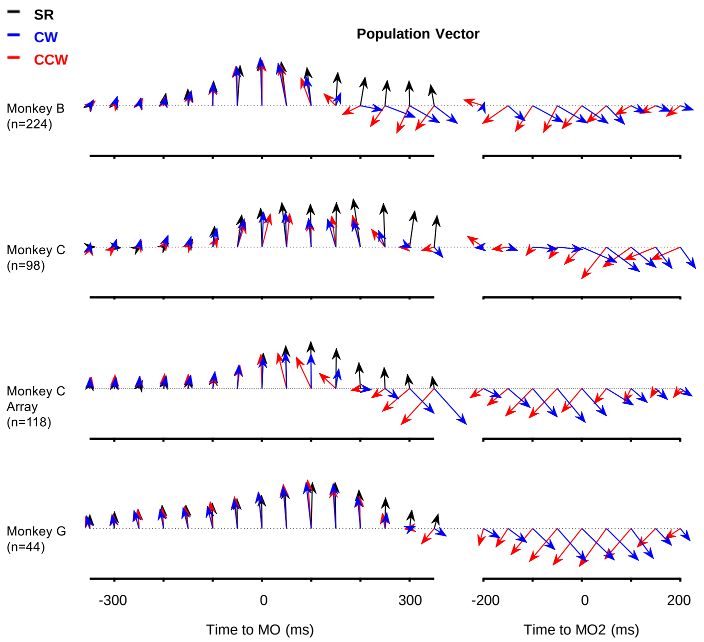

Supplementary Figure 8 Time series of population vectors.

Time-varying population vectors in SR (black), CW (blue), and CCW (red) conditions. The population vector in DR trials points to the same direction as in SR trials before the 1st reach onset, and then quickly shifts towards the 2nd reaching direction after the 1st movement onset. Therefore, the 1st movement direction can robustly be decoded via linear readout with single gain-modulated neurons.

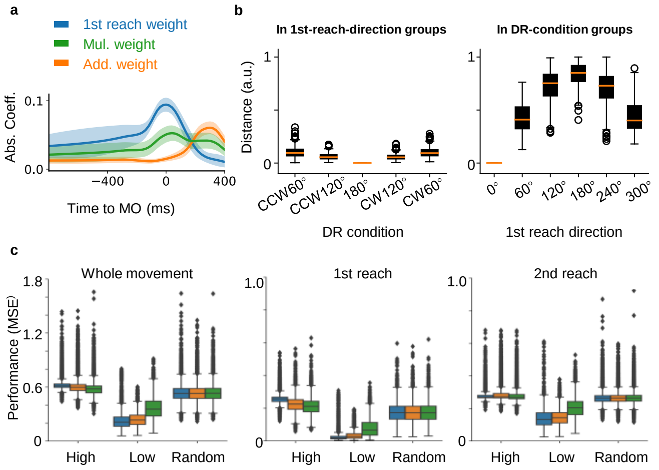

Supplementary Figure 9 Results of 100 networks with different random seeds.

a. Full model fitting result for RNN nodes (n=200*100), averaged over the mean of single networks. The error bar was plotted according to twice standard error. **b**. The distance of states under different DR conditions with states under 180° condition, within the 1st reach-direction clusters, along with the distance of states under different 1st reach conditions with states under 0° condition, within the DR-condition groups. Across 100 networks. **c**. The performance of networks without certain nodes during whole movement, 1st reach, and 2nd reach. High means those nodes with the highest 10% weights, Low means those nodes with the lowest 10% weights, and Random means randomly picked 10% of all activated nodes. Across 100 networks.

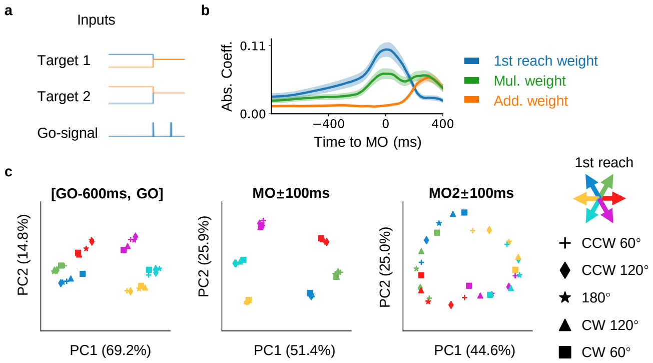

Supplementary Figure 10 Results of an RNN model with two triggers.

a. Schematic of the RNN model. Different from those with one trigger (Fig. 8) only from the Go-signal. While the first pulse located at GO, the second pulse located at 50ms before MO2. **b**. Full model fitting result for RNN nodes (n=200). The error bar was plotted according to twice standard error. **c**. The neural states obtained by PCA, forming an initial state spatial structure. Colors indicate the 1st movement directions; DR trials are presented in the same color family of related SR trials. Markers indicate 2nd reaching direction.

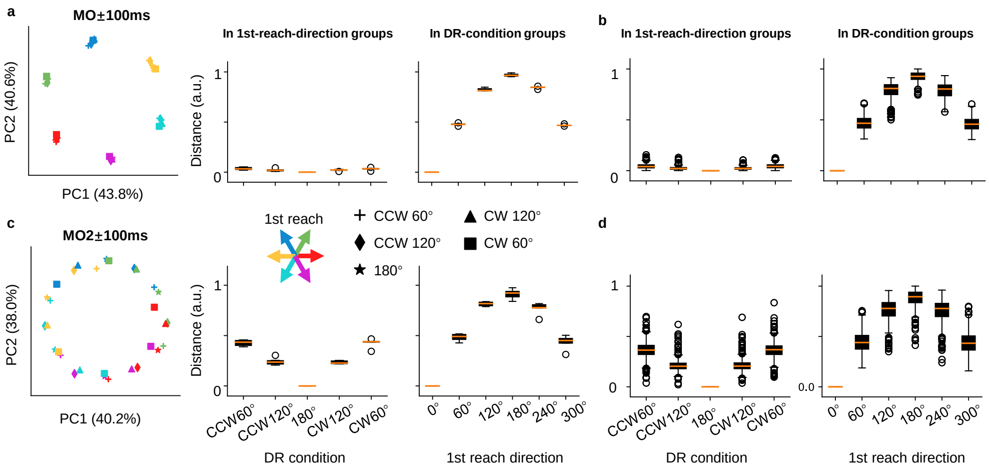

Supplementary Figure 11 Initial states distribution during execution.

a. Left: The PCA neural states indicating initial states; Right: the between distance, similar to Fig.8d right. Around 1st reach movement onset, for the example network. Colors indicate the 1st movement directions; DR trials are presented in the same color family of related SR trials. Markers indicate 2nd reaching direction. **b**. Similar with a, but for the 100 networks. **c**. The results around 2nd reach movement onset, for the example network. **d**. Similar with c, but for the 100 networks.
