# Supplementary material for: Multiplicative joint coding in preparatory activity for reaching sequence in macaque motor cortex
Source: Nat Commun. 2024 Apr 11;15:3153. doi: 10.1038/s41467-024-47511-1 (PMC11009282; doi:10.1038/s41467-024-47511-1)
Supplement: Supplementary file 3 — Reporting Summary [file 41467_2024_47511_MOESM3_ESM.pdf]

nature portfolio

Corresponding author(s): He Cui

Last updated by author(s): Feb 8, 2024

Reporting Summary

Nature Portfolio wishes to improve the reproducibility of the work that we publish. This form provides structure for consistency and transparency in reporting. For further information on Nature Portfolio policies, see our Editorial Policies and the Editorial Policy Checklist.

Statistics

For all statistical analyses, confirm that the following items are present in the figure legend, table legend, main text, or Methods section.

n/a	Confirmed	
☐	☒	The exact sample size (*n*) for each experimental group/condition, given as a discrete number and unit of measurement
☐	☒	A statement on whether measurements were taken from distinct samples or whether the same sample was measured repeatedly
☐	☒	The statistical test(s) used AND whether they are one- or two-sided<br>*Only common tests should be described solely by name; describe more complex techniques in the Methods section.*
☐	☒	A description of all covariates tested
☐	☒	A description of any assumptions or corrections, such as tests of normality and adjustment for multiple comparisons
☐	☒	A full description of the statistical parameters including central tendency (e.g. means) or other basic estimates (e.g. regression coefficient) AND variation (e.g. standard deviation) or associated estimates of uncertainty (e.g. confidence intervals)
☐	☒	For null hypothesis testing, the test statistic (e.g. *F*, *t*, *r*) with confidence intervals, effect sizes, degrees of freedom and *P* value noted<br>*Give P values as exact values whenever suitable.*
☒	☐	For Bayesian analysis, information on the choice of priors and Markov chain Monte Carlo settings
☒	☐	For hierarchical and complex designs, identification of the appropriate level for tests and full reporting of outcomes
☐	☒	Estimates of effect sizes (e.g. Cohen's *d*, Pearson's *r*), indicating how they were calculated

Our web collection on statistics for biologists contains articles on many of the points above.

Software and code

Policy information about availability of computer code

Data collection	The behavior task was controlled by custom code based on MonkeyLogic package, Matlab (R2011b). Blackrock Microsystems Neural Signal Processor and Central Suite (7.0.6) were used for array data collection and Alphalab and AlphaOmega SNR were used for single neuron recording.
Data analysis	Spike data were sorted off-line using Spike2 (v7) and wave_clus (v3.0). Analyses used custom code written in Matlab (R2021b) and Spyder (v5) with Python (v3.8). Recurrent neural network was trained using Pytroch (v1.10.0).

For manuscripts utilizing custom algorithms or software that are central to the research but not yet described in published literature, software must be made available to editors and reviewers. We strongly encourage code deposition in a community repository (e.g. GitHub). See the Nature Portfolio guidelines for submitting code & software for further information.

Data

Policy information about availability of data

All manuscripts must include a data availability statement. This statement should provide the following information, where applicable:

- Accession codes, unique identifiers, or web links for publicly available datasets
- A description of any restrictions on data availability
- For clinical datasets or third party data, please ensure that the statement adheres to our policy

The data used in this study are available at https://zenodo.org/doi/10.5281/zenodo.10637304.

Research involving human participants, their data, or biological material

Policy information about studies with human participants or human data. See also policy information about sex, gender (identity/presentation), and sexual orientation and race, ethnicity and racism.

Reporting on sex and gender	N/A
Reporting on race, ethnicity, or other socially relevant groupings	N/A
Population characteristics	N/A
Recruitment	N/A
Ethics oversight	N/A

Note that full information on the approval of the study protocol must also be provided in the manuscript.

Field-specific reporting

Please select the one below that is the best fit for your research. If you are not sure, read the appropriate sections before making your selection.

☒ Life sciences ☐ Behavioural & social sciences ☐ Ecological, evolutionary & environmental sciences

For a reference copy of the document with all sections, see nature.com/documents/nr-reporting-summary-flat.pdf

Life sciences study design

All studies must disclose on these points even when the disclosure is negative.

Sample size	We collected 279 and 117 units from monkey B and C through single electrode recording. We also collected 252 (reveiwer suggests to exclude neurons in PMd array, so the number becomes 169 in the manuscript) and 63 units from monkey C and G. No statistical methods were used to determine sample sizes, but our sample sizes are similar to those in previous publications (Parthasarathy, et al., 2017; Ames, et al., 2019; Zimnik and Churchland, 2021).
Data exclusions	Neurons with bad signal-to-noise ratio or very low firing rate (<1HZ) were excluded.
Replication	The experiments were carried out in 3 animals. All results were repeated successfully.
Randomization	N/A. No different experiment groups in the experiment. Task conditions were pseudo-randomly interleaved in each session.
Blinding	N/A. No experiment groups were assigned.

Reporting for specific materials, systems and methods

We require information from authors about some types of materials, experimental systems and methods used in many studies. Here, indicate whether each material, system or method listed is relevant to your study. If you are not sure if a list item applies to your research, read the appropriate section before selecting a response.

Materials & experimental systems

n/a	Involved in the study	
☒	☐	Antibodies
☒	☐	Eukaryotic cell lines
☒	☐	Palaeontology and archaeology
☐	☒	Animals and other organisms
☒	☐	Clinical data
☒	☐	Dual use research of concern
☒	☐	Plants

Methods

n/a	Involved in the study	
☒	☐	ChIP-seq
☒	☐	Flow cytometry
☒	☐	MRI-based neuroimaging

Animals and other research organisms

Policy information about studies involving animals; ARRIVE guidelines recommended for reporting animal research, and Sex and Gender in Research

Laboratory animals	Three adult (6-10 years old) male rhesus macaques (Macaca mulatta), weight between 7-13kg.
Wild animals	N/A
Reporting on sex	N/A. No sex effect in this work.
Field-collected samples	N/A
Ethics oversight	All procedures were in accordance with NIH guidelines and were approved by the Institutional Animal Care and Use Committee (IACUC) of Institute of Neuroscience, CAS

Note that full information on the approval of the study protocol must also be provided in the manuscript.

Plants

Seed stocks	N/A
Novel plant genotypes	N/A
Authentication	N/A
